# Supplementary material for: Regulation of the immune tolerance system determines the susceptibility to HLA-mediated abacavir-induced skin toxicity
Source: Commun Biol. 2021 Sep 28;4:1137. doi: 10.1038/s42003-021-02657-2 (PMC8479119; doi:10.1038/s42003-021-02657-2)
Supplement: Supplementary file 2 — Supplementary Information [file 42003_2021_2657_MOESM2_ESM.pdf]

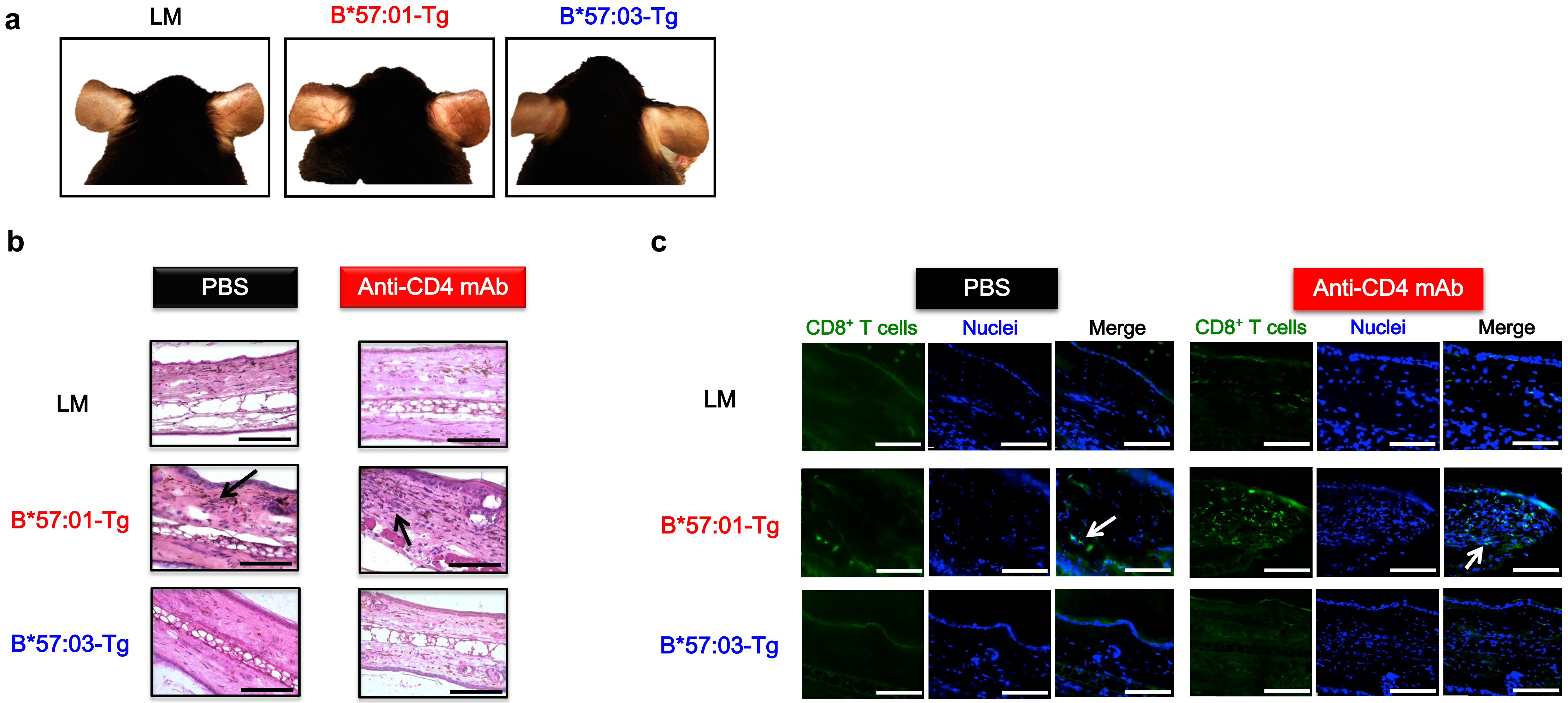

Supplementary Figure 1 | Effect of 3-week oral abacavir (ABC) administration plus application to the ear in $CD4^+$ T cell-depleted chimeric HLA transgenic mice

Representative images of the ears on day 21 **(a)**; ear sections either stained with hematoxylin and eosin (H&E) **(b)** or immunostained with anti-CD8 **(c)**, along with Hoechst 33342 nuclear staining. The ABC-fed B*57:01-Tg mice, B*57:03-Tg mice, and their littermates (LMs) were treated either with anti-CD4 monoclonal antibody ($CD4^+$ T cell depletion) or vehicle (PBS). All groups received oral ABC (1% *w/w*) plus ear painting (50 mg/kg/day) for 3 weeks. Each scale bar represents 100 μm. Arrows mark either the lymphocytic infiltration (H&E staining) or $CD8^+$ T cell infiltration (CD8 immunohistochemistry). Data are representative of two independent experiments.

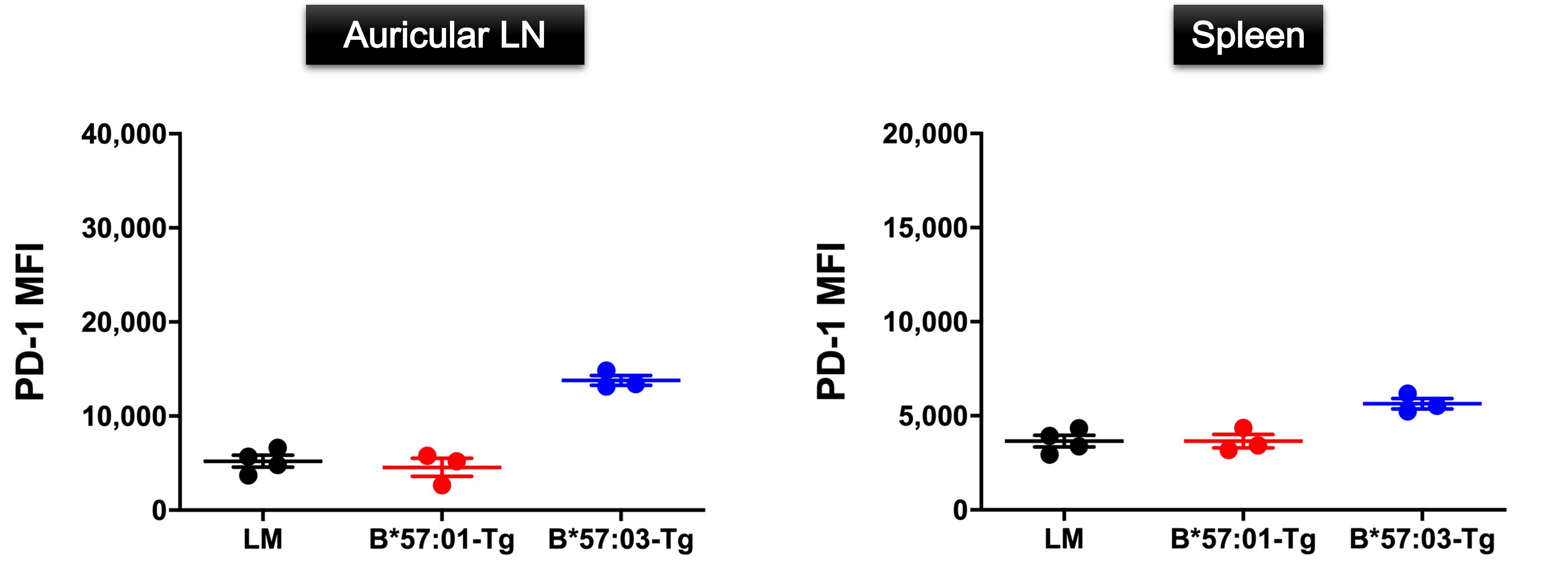

Supplementary Figure 2 | Effect of vehicle on PD-1 surface expression of effector memory $CD8^+$ T cells in $CD4^+$ T cell-depleted chimeric HLA transgenic mice

Flow cytometric measurement of median fluorescence intensity (MFI) values of PD-1 in effector memory $CD8^+$ T cells from auricular lymph node (LN) (left panel) or spleen (right panel) of $CD4^+$ T cell-depleted B*57:01-Tg mice, B*57:03-Tg, or littermates (LMs). The mice received a normal diet for 1 week. Effector memory $CD8^+$ T cells were gated from either lymphocytes or splenocytes by anti-CD44 antibody and anti-CD62L antibody (phenotype: $CD44^{high}CD62L^{low}$). Each plot represents an individual mouse with the mean ± SEM (N = 3). Data are summary of three independent experiments.

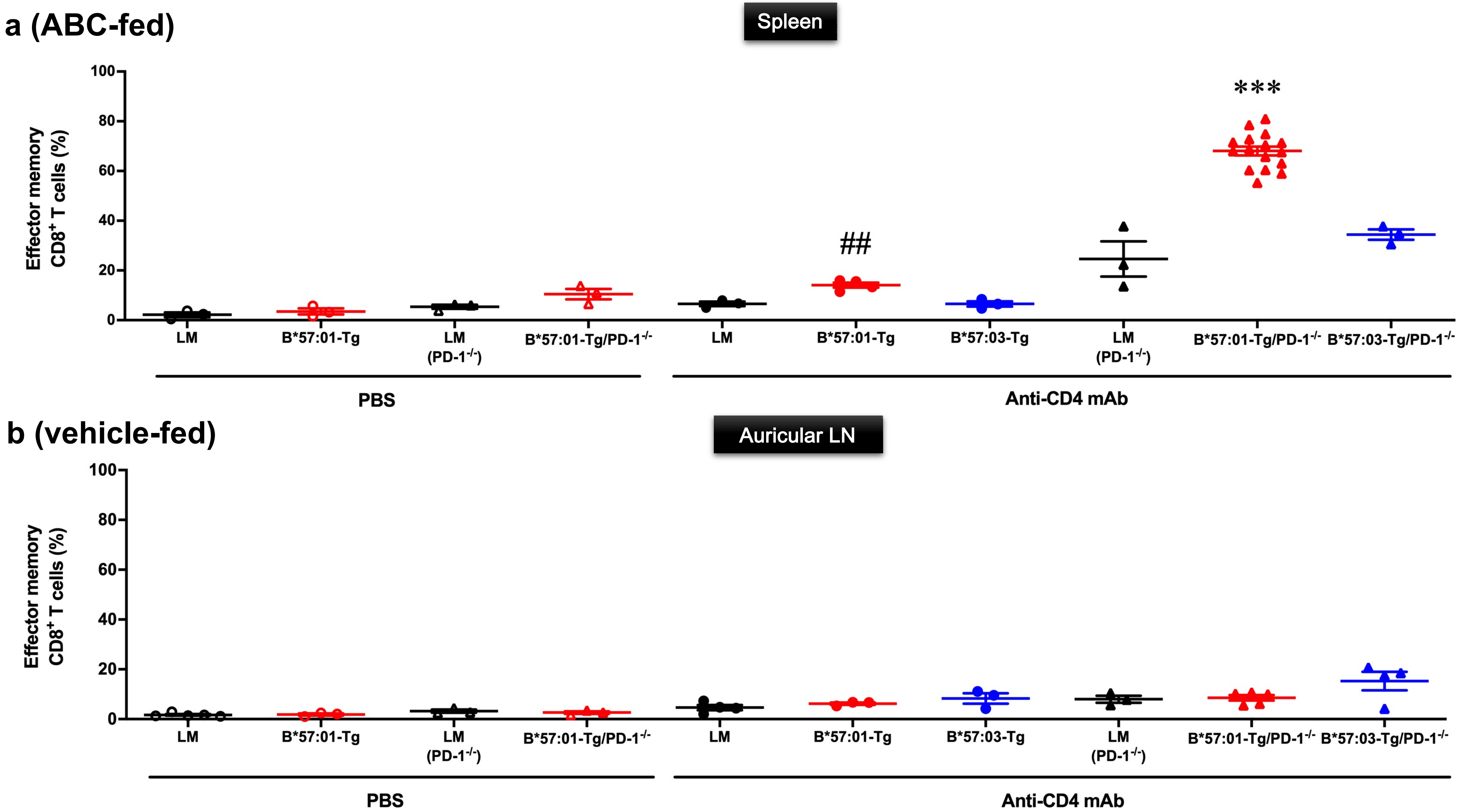

Supplementary Figure 3 | Effect of PD-1 knock out on ABC-induced activation of T cells in $CD4^+$ T cell-depleted chimeric HLA transgenic mice

(a-b) Percentages of effector memory T cells among total $CD8^+$ T cells isolated from (a) spleen or (b) auricular lymph node (LN). The mice received (a) 1% (*w/w*) ABC administered orally or (b) normal diet administrated for 1 week. Each plot represents an individual mouse with the mean ± SEM (N = 3-16); ***p < 0.001, compared with other mice groups; $^{\#\#}$p < 0.01, compared with $CD4^+$ T cell-depleted B*57:03-Tg mice or littermates (LMs), one-way ANOVA with Bonferroni's multiple comparisons correction. Data are summary of three independent experiments (except spleen of the $CD4^+$ T cell-depleted B*57:01-Tg/PD-$1^{-/-}$ mice, serving as the positive control per every independent experiment)

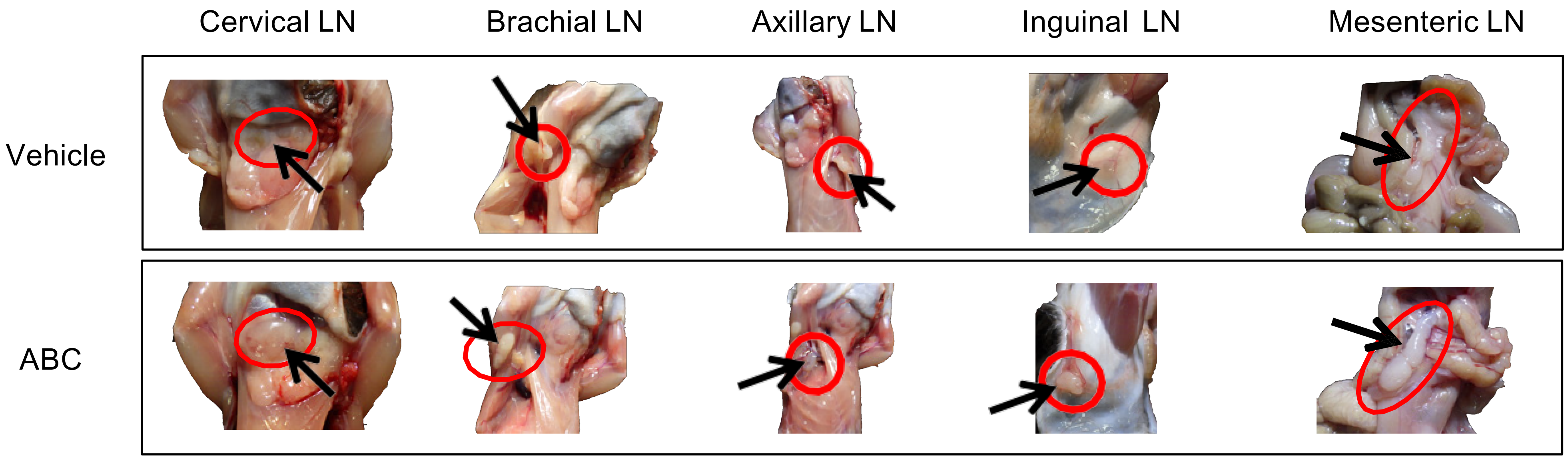

Supplementary Figure 4 | Effect of oral abacavir (ABC) in CD4^{+} T cell-depleted B*57:01-Tg/PD-1$^{-/-}$ mice
Representative images of photos of lymph nodes (LNs) in anti-CD4 mAb-treated B*57:01-Tg/PD-1$^{-/-}$ mice. Each group was orally administrated with 1% (*w/w*) abacavir (ABC) or a normal diet (vehicle) for 1 week.

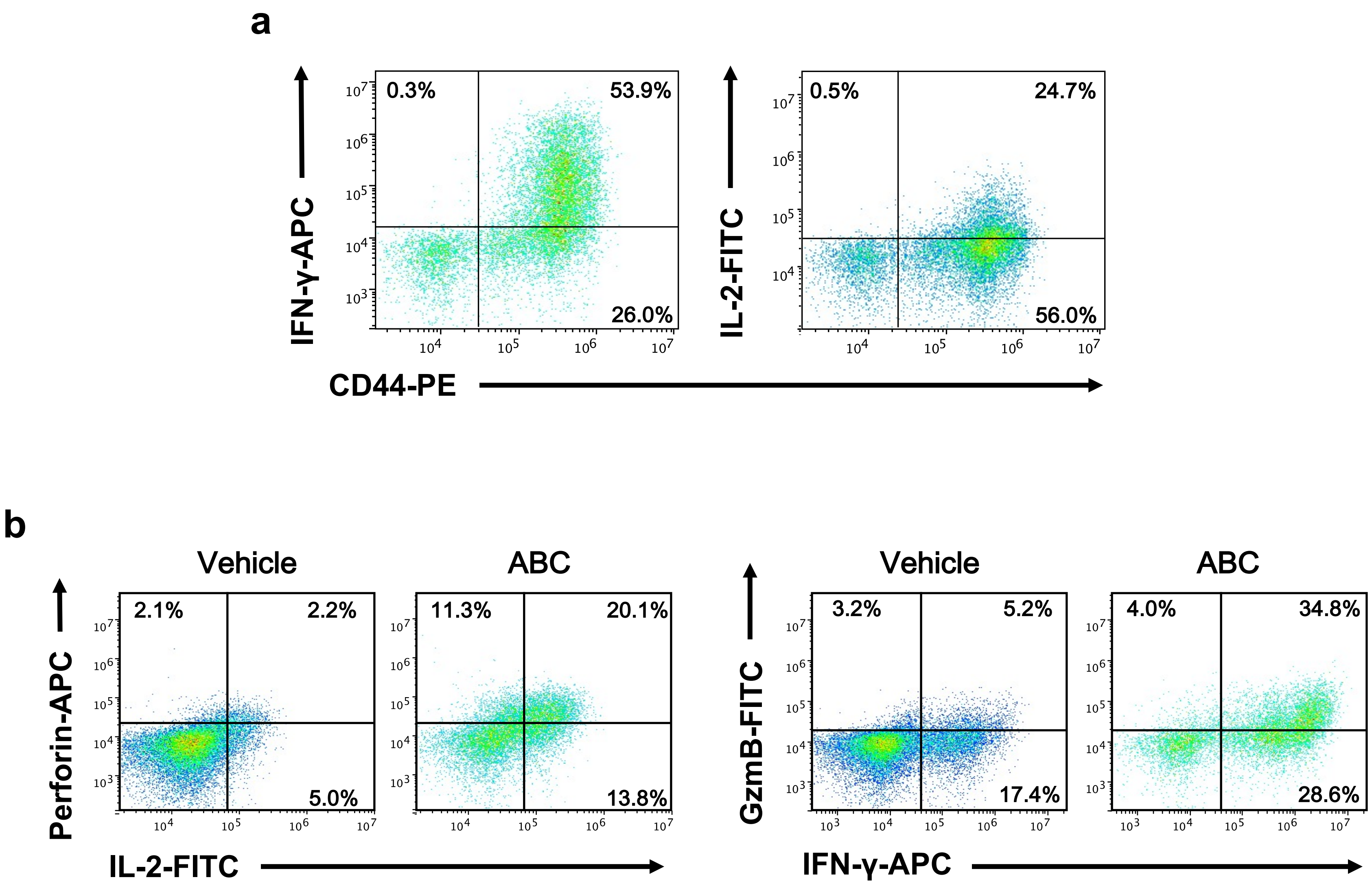

Supplementary Figure 5 | Cytokine and cytolytic granule production by CD8$^+$ T cells isolated from lymph nodes (LNs) of CD4$^+$ T cell-depleted B*57:01-Tg/PD-1$^{-/-}$ mice

(a) Representative dot plots depicting cytokine production by gated CD8$^+$ T cells of anti-CD4 monoclonal antibody-treated B*57:01-Tg/PD-1$^{-/-}$ mice. Mice received oral abacavir (ABC, 1% *w/w*) for 1 week. Data are representative of three independent experiments. **(b)** Representative dot plots depicting cytokine and cytolytic granule production in gated CD8$^+$ T cells of anti-CD4 mAb-treated B*57:01-Tg/PD-1$^{-/-}$ mice. Mice received oral abacavir (ABC, 1% *w/w*) or a normal diet (vehicle) for 1 week. Data are representative of three independent experiments.

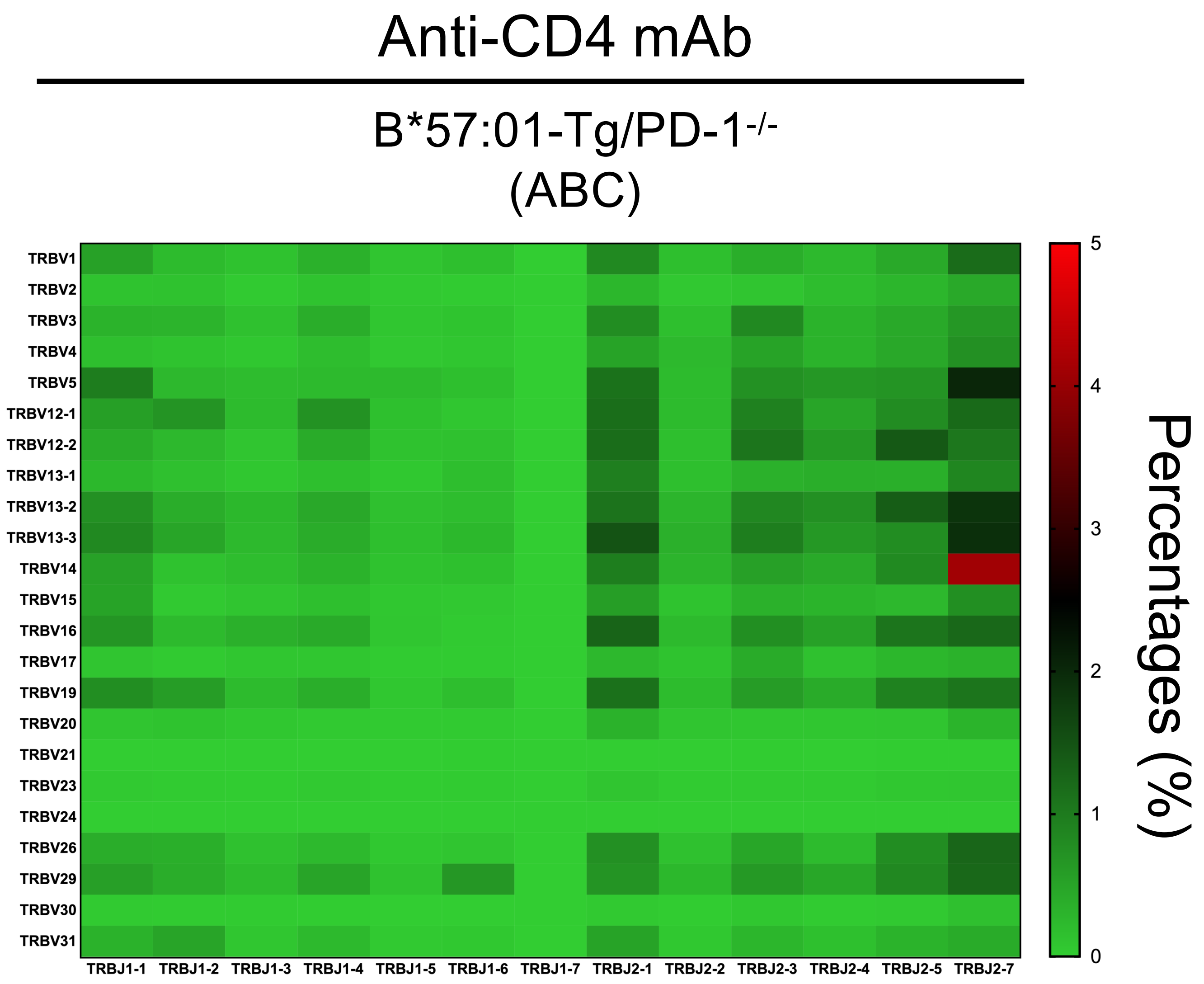

Supplementary Figure 6 | CD8$^+$ TCR repertoire in LNs of ABC-fed B*57:01-Tg/PD-1$^{-/-}$ mice

2D heat maps showing the percentages from low (0%) to high (5%) of Vβ and Jβ usage and combinations of productive sequences in total CD8$^+$ T cells. Data from two individual mice within a group of two independent experiments (N = 4) were collapsed prior to the analysis.

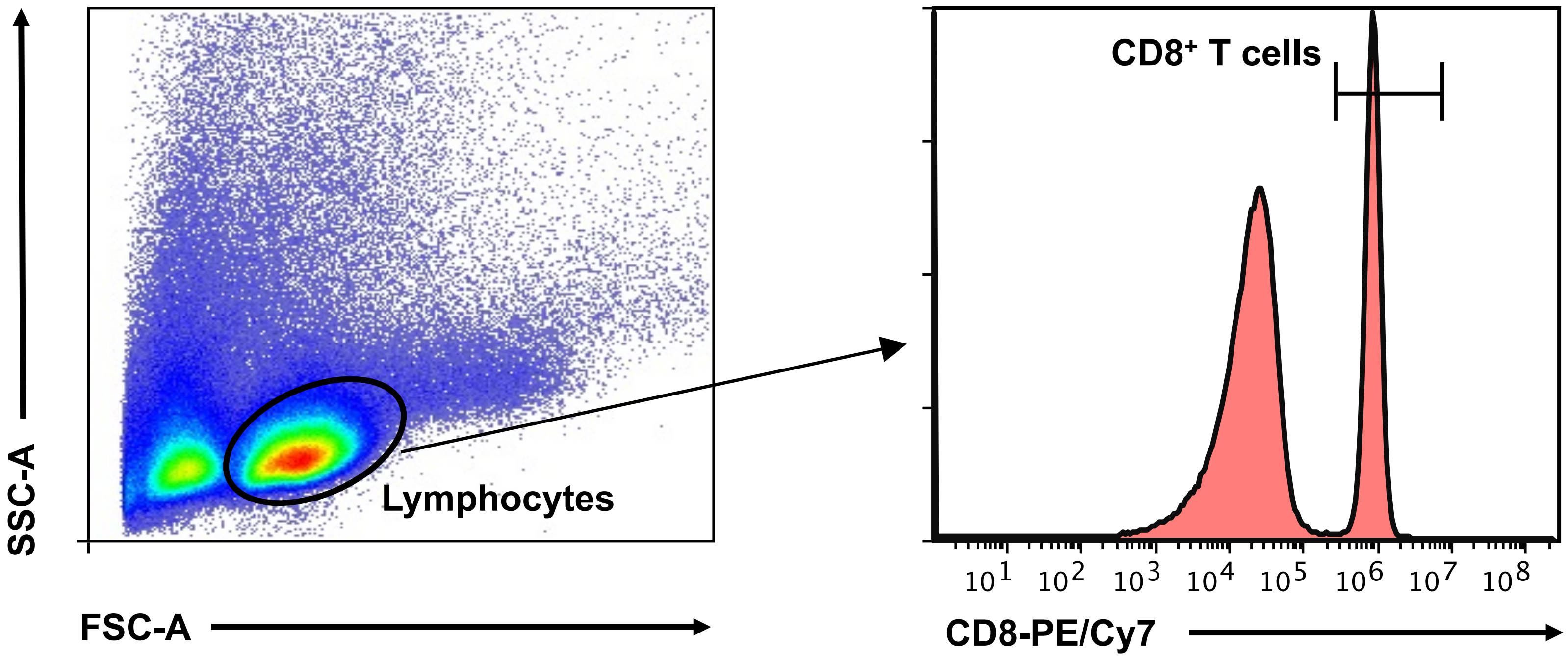

Supplementary Figure 7 | Gating strategies used for flow cytometry

Gating strategy for CD8^{+} T cells from lymph nodes and spleen for cell population analysis presented on Figs. 1b-c, 2a-b, 3a-c, and 5a-f, and Supplementary Figs. 2, 3, and 5.
