# Supplementary material for: Regulation of the immune tolerance system determines the susceptibility to HLA-mediated abacavir-induced skin toxicity
Source: Commun Biol. 2021 Sep 28;4:1137. doi: 10.1038/s42003-021-02657-2 (PMC8479119; doi:10.1038/s42003-021-02657-2)
Supplement: Supplementary file 5 — Reporting Summary [file 42003_2021_2657_MOESM5_ESM.pdf]

nature research

Corresponding author(s): Shigeki Aoki

Last updated by author(s): Aug 24, 2021

# Reporting Summary

Nature Research wishes to improve the reproducibility of the work that we publish. This form provides structure for consistency and transparency in reporting. For further information on Nature Research policies, see our Editorial Policies and the Editorial Policy Checklist.

## Statistics

For all statistical analyses, confirm that the following items are present in the figure legend, table legend, main text, or Methods section.

| n/a | Confirmed | |
|---|---|---|
| ☐ | ☒ | The exact sample size (*n*) for each experimental group/condition, given as a discrete number and unit of measurement |
| ☐ | ☒ | A statement on whether measurements were taken from distinct samples or whether the same sample was measured repeatedly |
| ☐ | ☒ | The statistical test(s) used AND whether they are one- or two-sided<br>*Only common tests should be described solely by name; describe more complex techniques in the Methods section.* |
| ☐ | ☒ | A description of all covariates tested |
| ☐ | ☒ | A description of any assumptions or corrections, such as tests of normality and adjustment for multiple comparisons |
| ☐ | ☒ | A full description of the statistical parameters including central tendency (e.g. means) or other basic estimates (e.g. regression coefficient) AND variation (e.g. standard deviation) or associated estimates of uncertainty (e.g. confidence intervals) |
| ☐ | ☒ | For null hypothesis testing, the test statistic (e.g. *F*, *t*, *r*) with confidence intervals, effect sizes, degrees of freedom and *P* value noted<br>*Give P values as exact values whenever suitable.* |
| ☒ | ☐ | For Bayesian analysis, information on the choice of priors and Markov chain Monte Carlo settings |
| ☒ | ☐ | For hierarchical and complex designs, identification of the appropriate level for tests and full reporting of outcomes |
| ☒ | ☐ | Estimates of effect sizes (e.g. Cohen's *d*, Pearson's *r*), indicating how they were calculated |

*Our web collection on statistics for biologists contains articles on many of the points above.*

## Software and code

Policy information about availability of computer code

| | |
|---|---|
| Data collection | Flow cytometry: EC800 cell analyzer (SONY)<br>Histological pictures: BZ-X700 microscope (Keyence)<br>PCR amplification and next generation sequencing (NGS) for the TCR repertoire: Illumina Miseq |
| Data analysis | Significant differences between the groups were analyzed using Bonferroni's test following 1-way ANOVA test.<br>Graphs except flowcytometric plots were produced using Graphpad Prism 8.<br>Flowlogic (Inivai Technologies) were used for producing flowcytometric plots.<br>TCR repertoire analysis was performed using Repertoire Genesis software. |

For manuscripts utilizing custom algorithms or software that are central to the research but not yet described in published literature, software must be made available to editors and reviewers. We strongly encourage code deposition in a community repository (e.g. GitHub). See the Nature Research guidelines for submitting code & software for further information.

## Data

Policy information about availability of data

All manuscripts must include a data availability statement. This statement should provide the following information, where applicable:

- Accession codes, unique identifiers, or web links for publicly available datasets
- A list of figures that have associated raw data
- A description of any restrictions on data availability

The source data of data presented in Figs. 1a, 1c, 2b, 3b, 4a-b, and 5c-f are provided as the Source Data file. The sequence data that support the findings of this study have been deposited at the DNA Data Bank of Japan (DDBJ) Sequence Read Archive (DRA) database under accession numbers DRA011282 and DRA011732. All other data are available from the authors upon reasonable request.

# Field-specific reporting

Please select the one below that is the best fit for your research. If you are not sure, read the appropriate sections before making your selection.

☒ Life sciences ☐ Behavioural & social sciences ☐ Ecological, evolutionary & environmental sciences

For a reference copy of the document with all sections, see nature.com/documents/nr-reporting-summary-flat.pdf

# Life sciences study design

All studies must disclose on these points even when the disclosure is negative.

| | |
|---|---|
| Sample size | No statistical measures were used to predetermine sample size. We typically performed N = 1-2 within each group of at least N ≥ 3 biological replicates for each experiment. |
| Data exclusions | Out-of-frame sequences were excluded from the T cell receptor repertoire analysis. |
| Replication | As reported in the Source Data file, the findings were reliably reproduced. |
| Randomization | Does not apply because the results of biochemical measurements are not affected by sample randomization. |
| Blinding | Does not apply because the results of biochemical measurements are not affected by knowledge of sample identities. |

# Reporting for specific materials, systems and methods

We require information from authors about some types of materials, experimental systems and methods used in many studies. Here, indicate whether each material, system or method listed is relevant to your study. If you are not sure if a list item applies to your research, read the appropriate section before selecting a response.

## Materials & experimental systems

| n/a | Involved in the study | |
|---|---|---|
| ☐ | ☒ | Antibodies |
| ☒ | ☐ | Eukaryotic cell lines |
| ☒ | ☐ | Palaeontology and archaeology |
| ☐ | ☒ | Animals and other organisms |
| ☒ | ☐ | Human research participants |
| ☒ | ☐ | Clinical data |
| ☒ | ☐ | Dual use research of concern |

## Methods

| n/a | Involved in the study | |
|---|---|---|
| ☒ | ☐ | ChIP-seq |
| ☐ | ☒ | Flow cytometry |
| ☒ | ☐ | MRI-based neuroimaging |

## Antibodies

Antibodies used

(BioLegend) anti-mouse-CD8a-PE/Cy7 (clone: 53-6.7)
(BioLegend) anti-mouse/humanCD44-PE (clone: IM7)
(BioLegend) anti-mouse-CD62L-FITC (clone: MEL-14)
(BioLegend) anti-human/mouse-Granzyme B-FITC (clone: GB11)
(BioLegend) anti-mouse-IFNγ-APC (clone: XMG1.2)
(BioLegend) anti-mouse-IL2-FITC (clone: JES6-5H4)
(BioLegend) anti-mouse-PD-1-APC (clone: RMP1-30)
(BioLegend) anti-mouse-Perforin-APC (clone: S16009A)
(BioLegend) anti-mouse-TNF-α-FITC (clone: MP6-XT22)
(Abcam) rat anti-mouse-CD8a (clone: YTS169.4)
(Abcam) goat anti-rat IgG H&L-Alexa Fluor® 488

Validation

All antibodies were commercially available, and specificity had been described by the manufacturer.

## Animals and other organisms

Policy information about studies involving animals; ARRIVE guidelines recommended for reporting animal research

| | |
|---|---|
| Laboratory animals | All experiments were conducted using the male mice in the C57BL/6 background, which were 8 – 16 weeks old. |
| Wild animals | The study did not involve wild animals. |

| | |
|---|---|
| Field-collected samples | Ear skin, Lymph nodes, Spleen |
| Ethics oversight | All animal procedures were approved by the Animal Care Committee of Chiba University (Chiba, Japan) (Animal Experiment Protocols: 2-332 and 3-87)). The animals were treated humanely in accordance with the guidelines issued by the National Institutes of Health (8th edition). |

Note that full information on the approval of the study protocol must also be provided in the manuscript.

# Flow Cytometry

## Plots

Confirm that:

☒ The axis labels state the marker and fluorochrome used (e.g. CD4-FITC).

☒ The axis scales are clearly visible. Include numbers along axes only for bottom left plot of group (a 'group' is an analysis of identical markers).

☒ All plots are contour plots with outliers or pseudocolor plots.

☒ A numerical value for number of cells or percentage (with statistics) is provided.

## Methodology

| | |
|---|---|
| Sample preparation | The cells were repsupend in 2%FBS/PBS, and then stained with different fluorescence-conjugated mAbs for 30 mins on ice. For the intracellular staining, cells were afterward fixed with 4% paraformaldehyde for 10 min at room temperature and permeabilized with 0.5% (v/v) Triton-X for 10 min at 4 °C. Finally, all samples were incubated with different fluorescence-conjugated mAbs for 30 min at 4 °C. |
| Instrument | EC800 cell analyzer (SONY) |
| Software | Flowlogic (Inivai Technologies) |
| Cell population abundance | No post-sort fractions were collected. |
| Gating strategy | The cells were gated on FSC-A/SSC-A to select the lymphocytes as the starting cell population, as exemplified in Supplementary Figure 7. |

☒ Tick this box to confirm that a figure exemplifying the gating strategy is provided in the Supplementary Information.
